# Reproducibility of peak force for isometric and isokinetic multi-joint leg extension exercise

**DOI:** 10.1186/s13102-025-01061-1

**Published:** 2025-01-29

**Authors:** Manfred Zöger, Alfred Nimmerichter, Arnold Baca, Klaus Wirth

**Affiliations:** 1 Training and Sports Sciences, University of Applied Sciences Wiener Neustadt, Johannes Gutenberg-Straße 3, Wiener Neustadt, 2700 Austria; 2 Centre for Sport Science and University Sports, University of Vienna, Auf der Schmelz 6, Wien, 1150 Austria; 3 Doctoral School of Pharmaceutical, Nutritional and Sport Sciences, University of Vienna, Josef-Holaubek-Platz 2, Wien, 1090 Austria

**Keywords:** Dynamometer, Isokinetic, Isomed 2000, Isometric, Leg press, Reproducibility

## Abstract

**Background:**

Isokinetic dynamometry is a common tool for evaluating muscle function and is used across various disciplines. Technical advancements have shifted focus towards multi-joint exercises such as the leg press, offering insights into practical human movement dynamics. However, previous reproducibility studies have focused predominantly on single-joint exercises, warranting investigations into the reliability of multi-joint exercises. This study aimed to assess the reproducibility of peak force (PF) during multi-joint leg press exercises using the IsoMed 2000 dynamometer.

**Methods:**

Thirty physically active subjects (mean: stature 179.4 cm; body mass 76.0 kg; age 30.6 years) participated in three testing sessions. Each session consisted of isometric and isokinetic leg press exercises. Knee angles for isometric exercises included 100° and 140°; velocities for isokinetic exercise included 30 mm/s and 600 mm/s. The first session served as the familiarization session. Statistical analysis included paired sample t-tests, Cohen’s d effect sizes, intraclass correlation coefficients (ICC), standard errors of measurement (SEM), and Bland-Altman calculations, including corresponding plots.

**Results:**

Descriptive data revealed consistent PF across sessions, with a significant between-sessions difference observed only for isometric (100°) leg extension in the right leg (*p* < 0.001; *d* = 0.13). ICC calculations showed high relative reproducibility (ICC > 0.911), with SEM ranging from 37.6 to 294.7 N (SEM% 2.3–6.3%, respectively). Bland-Altman plots depicted minimal intersession disparities (-141.8–68.3 N, respectively − 3.02–1.26%), supporting high reliability.

**Conclusions:**

This study highlights the reliability of assessing peak force during isometric and isokinetic leg press exercises using the IsoMed 2000 after a single familiarization session. These findings support its utility in muscular performance evaluation, urging practitioners to incorporate familiarization trials for accurate assessments.

## Introduction

The introduction of the isokinetic concept in the 1960s [1] marked a paradigm shift in muscle function assessment, with isokinetic dynamometry establishing itself as a widely accepted method for evaluating muscular performance [2–4]. Nowadays, isokinetic exercise, which is characterized by constant velocity movements against modulated resistance, has become an integral part of performance evaluation and training. Typical applications encompass a spectrum of disciplines within sport and medicine, including orthopedics, performance sports, rehabilitation, and sports physical therapy.

In the past, a majority of studies executing isokinetic dynamometry used single-joint exercises [5]. However, due to the technical development of multi-joint testing devices, there has been increasing interest in leg press exercises in recent years. The use of such devices enables the evaluation of muscular function within a multi-joint exercise, which is suggested to have greater practical relevance for actual everyday as well as sport-specific human movements than a single-joint exercise [6, 7]. For example, significant correlations were found for isokinetic leg press and several jumping exercises, as well as for squat and sprint performance [8–10]. The isokinetic multi-joint leg press exercise has therefore already been used for a variety of purposes, including the evaluation of different training and rehabilitation interventions [11–17]. In all of these settings, reliable data on muscular performance are paramount for optimizing training strategies, monitoring rehabilitation progress, and identifying potential performance limitations.

Previous studies on the topic of dynamometric reproducibility have focused mainly on single-joint exercises [5, 18–22], with only a handful investigating multi-joint leg press [23–26].

However, reproducibility results from a single-joint exercise cannot be transferred to a multi-joint exercise such as a leg press. It cannot be assumed that the generally high reliability of the knee joint automatically applies to other joints [27]. This is supported by the fact that only weak and non-significant correlations between single-joint and multi-joint isokinetic tests of the hip and knee extensors have been found [16, 28]. In general, reproducibility data should always be considered specific to the respective measurement device, test protocol, and subject group.

To the best of the authors’ knowledge, there is currently only one study examining the reproducibility of multi-joint leg extension exercises using the IsoMed 2000 device [23]. However, the authors used a different protocol in their study, investigating only moderate isokinetic velocities and no isometric contractions.

The aim of this study was, therefore, to assess the reproducibility of peak force (PF) for isometric and isokinetic multi-joint leg press exercise at two different isokinetic movement velocities (30 mm/s and 600 m/s) and at two different isometric knee angles (100° and 140°), using the IsoMed 2000 dynamometer.

## Methods

### Subjects

Thirty physically active subjects (25 male, 5 female; mean (SD): stature 179.4 (8.4) cm; body mass 76.0 (9.9) kg; age 30.6 (8.2) years) volunteered to participate in this study. An inclusion criterion for this study was the absence of any major previous orthopaedic lower extremity pathologies that would have needed clinical treatment. All subjects were physically active on a recreational level but did not have any previous experience in isokinetic exercise. Before starting the experiments, all participants received instructions to arrive at the laboratory in a mentally and physically rested state. Subjects were asked to ingest their last main meal at least 3 h before each test, to avoid any consumption of caffeine for 12 h, and to not engage in vigorous physical activity for 48 h before each test session. Written informed consent was provided by all subjects, and they were advised that withdrawal from the study is possible at any time. All subjects were fully informed about the experimental procedures and elucidated about the risks and benefits of participating in the study. The local research ethics board at the University of Applied Sciences Wiener Neustadt approved this study on the 5th of April 2021 (approval nr RB20210405013). All experiments conformed to the Declaration of Helsinki [29].

### Instruments

**Fig. 1:**
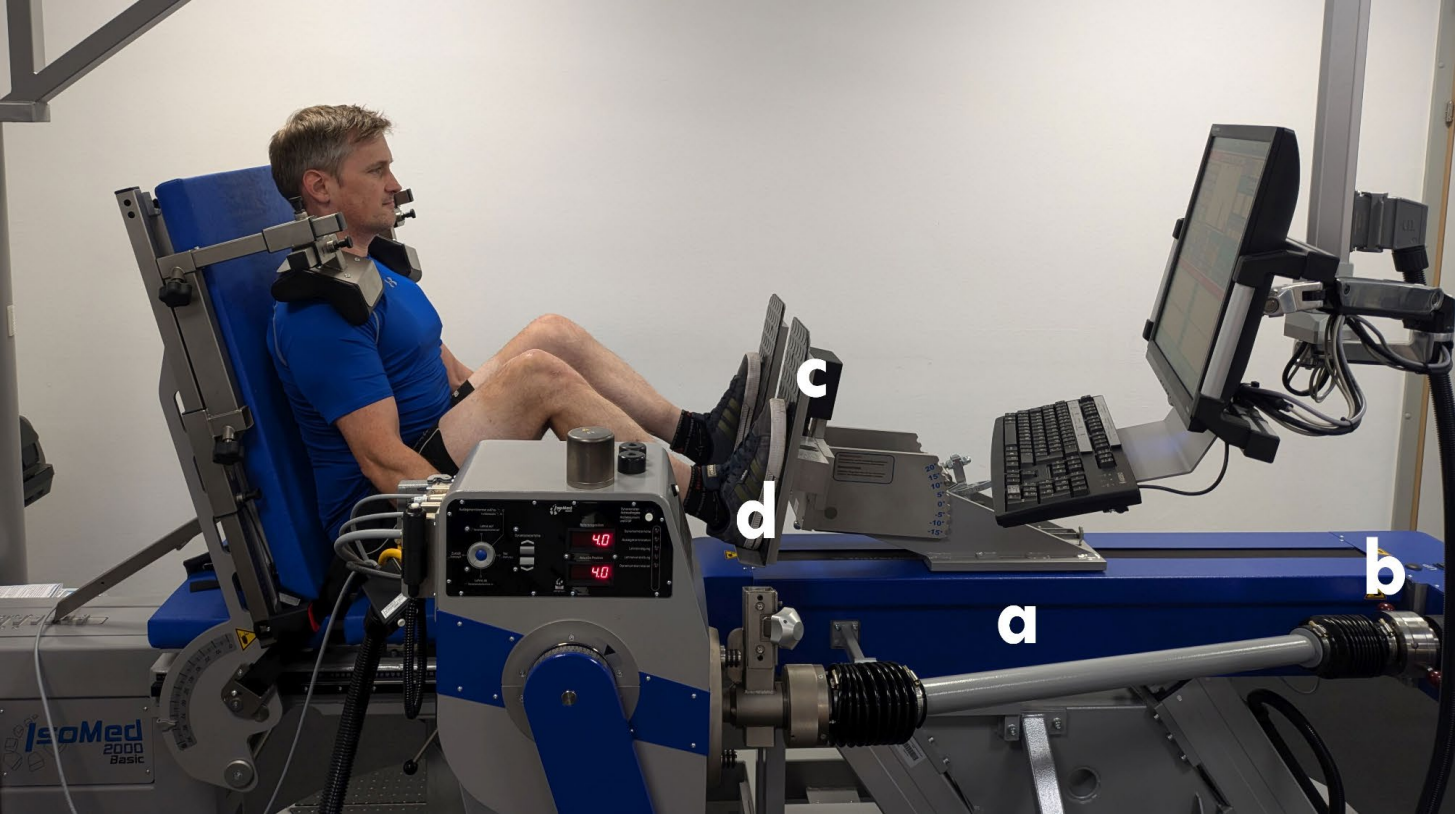
The IsoMed 2000 device with athletic linear module and a subject in the starting position. The IsoMed 2000 basic device converted to a motor-driven leg press using the manufacturers athletic linear module: **a**: drive shaft; **b**: selectable gears; **c**: force sensors; **d**: footrest

All experiments were conducted on an IsoMed 2000 dynamometer (D. & R. Ferstl GmbH, Hemau, Germany). The manufacturer provides different adaptors for this device, including a so-called athletic linear module that was used for this study. This module enables the conversion of the dynamometer into a motor-driven leg press (Fig. 1). A drive shaft (a) is used for converting the rotational motion of the dynamometer into a translational motion at the leg press via a tooth-belt linear drive. This drive shaft offers two gears (b) that differ in their ratio (gear I = 1:1; gear II = 1:1.5) and therefore determines the maximum linear movement speed (800 mm/s vs. 1,200 mm/s) and the maximum force (8,850 N vs. 5,850 N). Strain gauge force sensors (c) that are located at the back of the footrest (d) are used for the measurement of the applied force separately for the left and right legs.

For the present study, gear I of the drive shaft was used. Before each session, the device was calibrated according to the manufacturer’s instructions. Data recording was performed at a sample rate of 200 Hz using the manufacturer’s integrated computer software IsoMed Analyse SP3-i51.

### Procedures

Subjects were tested in three identical sessions. The first session was used as a familiarization trial to accommodate participants to the isokinetic device and protocol, as recommended in several studies [30–33]. Therefore, data from this session were not included in subsequent analyses. The sessions were typically scheduled 72–96 h apart to permit sufficient recovery for the subjects. Sessions were conducted at the same time of day (± 1.5 h) for each subject to minimize possible influences from diurnal variations. In an effort to minimize possible inter-tester variability, all tests in this study were conducted by the same examiner, who is a trained sport- and exercise scientist. Subjects were asked to arrive at the laboratory with shoes that had a hard rubber outsole without additional cushioning elements and to wear the same shoes for all sessions.

Each session started with a 10-min general warm-up, followed by the initial positioning of the subject on the device. The vertical backrest of the dynamometer was reclined to 75°, and the footrest was set at an inclination of 10° towards plantar flexion of the ankle. For fixation of the subject to the device and to minimize errant body movements, adjustable seat belts and pads were firmly applied, including the shoulders, thorax, and hip. In addition, the subjects were instructed to grasp the side handles of the device with their hands.

The range of motion and velocity served as the basic input parameters [34]. A mechanical handheld goniometer was utilized to set the range of motion to 90–170° (180° = fully extended). To do so, the trochanter major, lateral femoral epicondyle, and lateral malleolus were used as bony reference points and detected by palpation. The start and end of movement were measured while contracting the implicated muscles. For safety reasons and to minimize any risk of injuries, the popliteal pad of the device was adjusted and placed under the popliteal fossa of the subject in a way that prevented a complete extension (or even hyperextension) of the knee joint.

The subjects were then asked to put the leg onto the leg press footrest so that the heel ended up on the heel support area of the device. For isometric measurements, the footrest was fixed to achieve knee joint angles of 100° (Iso100) and 140° (Iso140). The position of the footrest used to achieve these angles was individually adjusted for each subject using the goniometer and the methods mentioned before.

Integrated software was used to record individual settings for each subject to guarantee identical positioning in every session. In each session, subjects performed isometric and isokinetic leg extensions in two conditions each. The conditions included isometric measurements with knee angles of 100° (Iso100) and 140° (Iso140), and isokinetic measurements that were conducted at translational velocities of 30 mm/s and 600 mm/s. All subjects performed each condition in three different ways: left-leg only (L), right-leg only (R), and two-legged (T). Subjects always started with T, followed by both one-legged conditions. Starting the one-legged conditions with the left or right leg was randomly assigned, although starting with the left vs. starting with the right leg was evenly distributed within the subject group.

The test order was Iso100, followed by Iso140 for isometric conditions. Isokinetic conditions were set at 30 mm/s, followed by 600 mm/s [35, 36]. The test order remained unchanged throughout all sessions. The initial starting position was achieved passively, and all isokinetic measurements were completed as discrete movements in a single direction [37]. To prepare for each condition of the test, subjects engaged in a submaximal specific warm-up on the device to familiarize themselves with the demands of each assessment. This specific warm-up comprised 10 repetitions at an intensity approximating 50% of the maximum voluntary contraction, followed by 3 repetitions at an intensity approximating 80% of the maximum voluntary contraction. Upon completion of this warm-up, subjects received a 3-min break that was used to repeat the explanation of the procedures for the subsequent condition using standardized instructions. Participants performed a minimum of three repetitions for each test condition. However, additional repetitions were performed until PF started to decline. Within a maximum of five repetitions, PF was achieved in all subjects. Before each repetition, participants received 3-min of passive rest to ensure sufficient recovery. Strong verbal encouragement from the examiner and visual feedback on a screen were provided to maximize the effort of the subjects.

### Statistical analysis

Two subjects failed to complete the entirety of the scheduled sessions. Consequently, their data were excluded, and subsequent analytical procedures were conducted on the remaining dataset, comprising 28 subjects. The main output parameter that was used for further analysis was the PF, as this parameter is commonly used in numerous studies dealing with muscular performance and dynamometers [34, 38]. For each condition, the repetition with the highest PF value was extracted from each session and subjected to analysis. Descriptive data are presented as means (± standard deviation). The normality of the data distribution was assessed using the Shapiro-Wilk test. To assess possible differences between PF measurements, paired sample t-tests were applied. The magnitude of the difference between sessions was calculated using Cohen’s *d* effect sizes, applying the formula: $\:\left(Mea{n}_{Session2}-Mea{n}_{Session3}\right)/SDpooled$. These were interpreted according to Cohen [39], where |*d*|0.2 = small, |*d*|0.5 = moderate, and |*d*|0.8 = large. Relative reproducibility was evaluated using the two-way random effect intraclass correlation coefficient (ICC) and interpreted by Vincent’s criteria [40]. According to these guidelines, an ICC exceeding 0.9 is considered high, while values between 0.8 and 0.9 are deemed moderate, and those below 0.8 are considered low. Absolute reproducibility was evaluated by calculating the standard error of measurement (SEM) using the formula $\:SEM=SD\:\times\:\:\sqrt{\:1-ICC}$ [41, 42]. Additionally, the SEM% was computed, defined as SEM/(means of measurements from session) * 100. To quantify the level of agreement between sessions, Bland-Altman statistics ± 95% limits of agreement (LoA) were calculated, and corresponding plots were generated to visually depict individual results [43]. Statistical analyses were conducted employing IBM SPSS Statistics for Windows, version 28.0 (IBM Corp., Armonk, NY, USA). Figures were created using GraphPad Prism V.9.3 for Windows (GraphPad Software, San Diego, CA, USA). The threshold for statistical significance was set at *p* < 0.05.

## Results

PF was significantly higher (+ 44.2 N) during the third session for Iso100 in the right leg condition (t(27)= -3.69, *p* = < 0.001; *d* = 0.13). No other significant differences in PF between sessions were observed. For comparison of sessions, small effect sizes in the range of *d* < 0.01 to 0.14 were found (Table 1).

The ICC results indicate high relative reproducibility, with values ranging from 0.911 to 0.989 (95% CI 0.809 to 0.995). The absolute reproducibility was expressed as SEM and SEM% and revealed values from 37.6 to 294.7 N and 2.3 to 6.3%, respectively (Table 2).

**Fig. 2:**
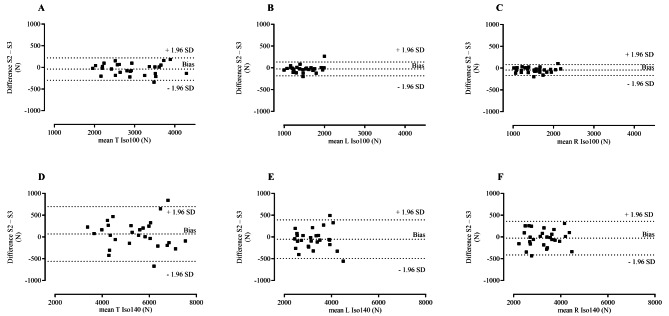
Bland-Altman plots– differences between isometric session two and session three plotted against the means. Differences between session two and session three plotted against the means of session two and session three for (**A**) two-legged isometric extension at 100° knee angle; (**B**) left leg only isometric extension at 100° knee angle; (**C**) right leg only isometric extension at 100° knee angle; (**D**) two-legged isometric extension at 140° knee angle; (**E**) left leg only isometric extension at 140° knee-angle; (**F**) right leg only isometric extension at 140° knee angle

**Fig. 3:**
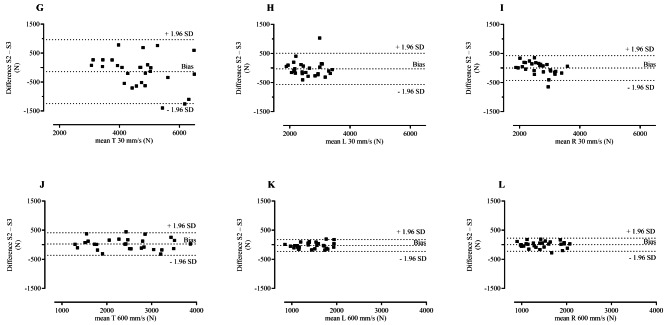
Bland-Altman plots– differences between isokinetic session two and session three plotted against the means. Differences between session two and session three plotted against the means of session two and session three for (**G**) two-legged isokinetic extension at 30 mm/s; (**H**) left leg only isokinetic extension at 30 mm/s; (**I**) right leg only isokinetic extension at 30 mm/s; (**J**) two-legged isokinetic extension at 600 mm/s; (**K**) left leg only isokinetic extension at 600 mm/s; (**L**) right leg only isokinetic extension at 600 mm/s

Bland-Altman plots illustrate a stochastic relationship for isometric and isokinetic leg extension between subject-specific differences and session averages (Figs. 2 and 3). The bias, which represents the average difference between sessions (Table 3), ranged from − 141.8 to 68.3 N (95% LoA from − 1246.0 to 962.4), where a negative value indicates that session three had a greater value than session two.

**Table 1:** Mean (SD) for peak force measurements as well as *p*-values and cohens’ *d* effect sizes for comparison of sessions

	Session 2 (*N*)	Session 3 (*N*)	pvalue	Effect size
T Iso100 L Iso100	2920.5 (633.0) 1480.4 (300.9)	2959.5 (641.9) 1506.4 (283.6)	0.127 0.100	0.06 0.09
R Iso100 *	1511.5 (333.0)	1555.8 (330.6)	< 0.001	0.13
T Iso140 L Iso140 R Iso140 T 30 mm/s L 30 mm/s R 30 mm/s	5461.4 (1130.5) 3219.0 (609.0) 3250.7 (637.7) 4630.4 (893.8) 2585.2 (472.9) 2648.4 (414.7)	5393.1 (1157.1) 3271.0 (613.7) 3278.4 (630.4) 4772.1 (1085.8) 2615.0 (479.7) 2650.5 (513.8)	0.269 0.234 0.464 0.194 0.569 0.960	0.06 0.09 0.04 0.14 0.06 < 0.01
T 600 mm/s L 600 mm/s R 600 mm/s	2461.4 (722.4) 1425.5 (332.1) 1446.6 (360.4)	2438.2 (738.4) 1452.8 (316.0) 1445.9 (368.7)	0.539 0.178 0.974	0.03 0.08 < 0.01

N– Newton, T– two-legged, R– right leg only, L– left leg only, Iso100– isometric 100° knee angle, Iso140– isometric 140° knee angle

* significant difference between session 2 and session 3 at *p* < 0.001

**Table 2:** Relative and absolute reproducibility statistics for comparison of peak force from sessions 2 and 3

	ICC (2,1)	95% CI	SEM (*N*)	SEM (% of mean)
T Iso100 L Iso100	0.989 0.979	0.978–0.995 0.955–0.990	66.3 42.0	2.3 2.8
R Iso100 T Iso140 L Iso140	0.987 0.980 0.964	0.948–0.995 0.957–0.991 0.923–0.983	37.6 160.4 115.0	2.4 5.5 3.5
R Iso140	0.976	0.948–0.989	97.4	3.0
T 30 mm/s L 30 mm/s R 30 mm/s T 600 mm/s L 600 mm/s R 600 mm/s	0.911 0.912 0.945 0.982 0.972 0.975	0.809–0.958 0.811–0.959 0.880–0.974 0.961–0.992 0.941–0.987 0.947–0.989	294.7 140.1 108.5 97.1 53.8 57.1	6.3 5.4 4.1 4.0 3.7 3.9

ICC– intraclass correlation coefficient, CI– confidence interval, SEM– standard error of measurement, N– Newton, T– two-legged, L– left leg only, R– right leg only, Iso100– isometric 100° knee angle, Iso140– isometric 140° knee angle

**Table 3:** Bias (SD) in Newton and % as well as 95% LoA for comparison of sessions 2 and 3 received from Bland-Altman calculations

	Bias (*N*)	Bias (%)	95% LoA (*N*)
T Iso100 L Iso100	-39.1 (131.4) -26.0 (80.7)	-1.33 -1.74	-296.6–218.5 -184.2–132.2
R Iso100 T Iso140 L Iso140	-44.2 (63.4) 68.3 (320.1) -52.0 (226.1)	-2.88 1.26 -1.60	-168.4–79.9 -559.0–695.6 495.1–391.0
R Iso140	-27.7 (197.4)	-0.85	-414.7–359.3
T 30 mm/s L 30 mm/s R 30 mm/s T 600 mm/s L 600 mm/s R 600 mm/s	-141.8 (563.3) -29.8 (273.5) -2.1 (217.4) 23.2 (197.3) -27.3 (104.5) 0.7 (114.8)	-3.02 -1.15 -0.08 0.95 -1.90 0.05	-1246.0–962.4 -565.8–506.3 -428.2–424 -363.4–409.9 -232.2–177.6 -224.4–225.8

LoA– limits of agreement, N– Newton, T– two-legged, L– left leg only, R– right leg only, Iso100– isometric 100° knee angle, Iso140– isometric 140° knee angle

## Discussion and implications

This study aimed to determine the reproducibility of PF during maximum isometric and isokinetic multi-joint leg extension exercises using the IsoMed 2000 dynamometer with a corresponding athletic linear module. In general, the results of this study indicate a high reliability of PF after initial familiarization.

It is a characteristic of isokinetic leg press exercise using a dynamometer such as the IsoMed 2000 that a constant linear velocity of the device’s footrest does not result in constant joint angle velocity throughout the range of motion. In addition, the same linear velocity can result in different angular velocities for subjects with different statures and/or segment lengths [34]. In an attempt to overcome these limitations, a correction procedure has been developed [6]. However, this mathematical model and the underlying assumptions are based on simplified principles that, in practice, only apply to a limited extent. A recent study [44] showed that this procedure is not free of errors and can significantly differ from marker-derived metrics. Due to these concerns and the fact that we controlled for all other possible input parameters (e.g., range of motion, exact placement of subjects), we decided not to apply the correction procedure and instead opted for constant linear velocity throughout all subjects.

Regarding inter-session disparity, no significant differences were found between sessions two and three, except for Iso100 in the right leg. With Cohen’s *d* effect sizes in the range of < 0.01 to 0.14, all of these differences appear to be small.

Concerning reproducibility, we observed ICC values > 0.911 with a 95% CI of 0.809 or greater. The lowest ICCs were found for slow isokinetic velocities. However, as all ICCs are > 0.9, the relative reproducibility can generally be rated as high for all conditions, in accordance with the recommendations of Vincent [40]. Corresponding SEM measurements of 37.6–294.7 N, or 2.3–6.3%, can be considered appropriate for the majority of common practical applications.

Comparing our ICC results to those of other studies, these results are similar [24–26, 45] or greater [46, 47] than those of previous investigations on peak force reproducibility in multi-joint leg extension. However, all these studies used a different device than the one used in our study. The only experiment conducted using the IsoMed 2000 [23] revealed similar results for the ICCs when comparing session two and session three. However, Dirnberger, Huber [23] used a slightly different methodological approach and tested only one leg.

With regard to SEM% results, we observed 2.3 to 6.3% which is lower than in the study of Müller, Baur [46], who found distinctly higher values, reaching 9.5 to 10%, although the authors did not implement a familiarization trial in their study.

In general, our results reveal lower reliability (absolute and relative) for the slower isokinetic velocity in comparison to the faster one. This trend for lower reliability at slower velocities is in agreement with the results of Davies and Heiderscheit [24] and Levine, Klein [48]. According to Levine, Klein [48] the longer contraction time and higher force at slower velocities could lead to a slightly altered position of the subjects on the device. To address this, in our study, we opted to minimize errant body movements using straps and pads. Because of this and that there was only a minimum difference in the magnitude of ICC and SEM between slow and fast velocities, our data suggest that velocity did not affect the results of our study.

The systematic difference, or bias, representing the average disparity between sessions varied from − 141.8 to 68.3 N. Notably, a negative value signifies that session three exhibited a higher average value than did session two. In our study, nine out of twelve conditions revealed a negative bias, illustrating the importance of at least one familiarization trial. However, considering the relative bias (-3.02 to 1.26%), this finding supports the high reliability of the measurement protocol in this study.

### Conclusion

This study assessed the reliability of PF measured during various isometric and isokinetic leg press extension exercises, utilizing the IsoMed 2000 dynamometer. The results indicate that using this methodology, PF measurements achieve a level of reliability deemed excellent for most common practical applications. Therefore, professionals are encouraged to incorporate at least one familiarization trial for each chosen angle and velocity when implementing isometric or isokinetic testing, ensuring the validity of subsequent results.

As the isokinetic leg press strength already showed correlations with jump-, squat-, and sprint performance in previous studies and is frequently used for training and rehabilitation interventions, our results could provide valuable information for practitioners when interpreting diagnostic data.

## Data Availability

The datasets used and analysed during the current study are available from the corresponding author upon reasonable request.

## Abbreviations

ICC: Intraclass correlation coefficient
Iso100: Isometric 100° knee angle
Iso140: Isometric 140° knee angle
L: Left-leg only
LoA: Limits of agreement
N: Newton
PF: Peak force
R: Right-leg only
SEM: Standard error of measurement
T: Two-legged
